# Analysis of Volatile Compounds and Sugar Content in Three Polish Regional Ciders with Pear Addition

**DOI:** 10.3390/molecules25163564

**Published:** 2020-08-05

**Authors:** Jarosław Kliks, Joanna Kawa-Rygielska, Alan Gasiński, Adam Głowacki, Antoni Szumny

**Affiliations:** 1 Department of Human Nutrition and Diet Therapy, Branch Faculty of University of Zielona Góra in Sulechów, ul. Armii Krajowej 51, 66-100 Sulechów, Poland; j.kliks@wzs.uz.zgora.pl; 2 Department of Fermentation and Cereals Technology, Faculty of Biotechnology and Food Science, Wrocław University of Environmental and Life Science, Chełmońskiego 37 Street, 51-630 Wrocław, Poland; alan.gasinski@upwr.edu.pl (A.G.); adamglowackigag@gmail.com (A.G.); 3 Department of Chemistry, Faculty of Biotechnology and Food Science, Wrocław University of Environmental and Life Sciences, C.K. Norwida Street 25, 50-375 Wrocław, Poland; antoni.szumny@upwr.edu.pl

**Keywords:** ciders, volatile compounds, gas chromatography, mass spectrometry, apples, pears

## Abstract

Aroma plays important part in cider acceptability to the consumer. In this study, techniques such as headspace solid-phase microextraction (HS-SPME), which has been coupled with gas chromatography and mass spectrometry, have been used to assess what changes in the volatilome occur during fermentation of three apple cultivars (*Cortland, Gala, Idared*) with and without addition of pear (*Konferencja)* juice addition. Analysis of volatiles has shown that temperature of fermentation, apple variety and pear juice addition have significant influences on the volatile compositions of the acquired ciders. Ciders prepared in laboratory conditions fermented at 15 °C were characterized by a greater share of esters, such as ethyl hexanoate, ethyl decanoate and ethyl dodecanoate, in volatile profile (66.24–79.58%) than ciders fermented at 20 °C (58.81–77.22%). Ciders fermented at a higher temperature were characterized by a greater share of alcohols, such as phenylethyl alcohol and hexan-1-ol (18.34–36.7%) than ciders fermented at a lower temperature (16.07–25.35%). In the ciders prepared from pear (20% *w/w*) and apple (80% *w/w*) juice, the presence of esters, such as ethyl (2*E*, 4*Z*)-deca-2,4-dienoate, characterized by a pear aroma, could be noted.

## 1. Introduction

Cider (also called “hard cider,” in North America) is an alcoholic drink obtained by alcoholic fermentation of apple juice, although many ciders nowadays are produced from diluted apple juice concentrate. Cider can be consumed as a final product, albeit it can be also used as a substrate for the production of cider vinegar, produced by acetic acid bacteria or by the means of distillation, for the production of apple brandies, such as Calvados in Normandy and Lambig in Brittany. Cider is currently produced in many countries on the North American, Asian, European and Australian continents, which is why its individual types may differ significantly, depending on the region or country wherein they are produced [1]. In Poland, ciders are made by alcoholic fermentation of an apple juice or diluted apple juice concentrate with the possibility of adding pear juice in an amount of up to 40% by volume [2]. Apples and pears are the sources of many phytochemicals and vitamins, which are beneficial for human health, but health-promoting properties of the cider are not the most important ingredients which have impacts on the aroma of the cider [3,4,5,6,7]. Volatile compounds play a crucial part in creating the fragrance of the cider and other alcoholic beverages [8]. There are many factors which influence these characteristics, such as the variety of apple used, the degree of fruit ripeness, the conditions under which apple trees are grown, the processes used to extract apple juice and the fermentation temperature and species of microorganisms used for fermentation [9]. The main chemical groups of volatile compounds identified in cider are alcohols, acids and esters, such as 1-butanol, 1-hexanol, 3-methylbutyl acetate, 2-phenylethyl acetate and butyl acetate naturally occurring in fruits and produced by microorganisms that are used to ferment cider [10]. Volatile compounds derived from ciders have been studied by many scientists, but there still exists a gap in knowledge about the impact of the addition of pears or pear juice on the aroma of cider [11]. These studies are important, among others, because Polish legislation does not allow for pear fragrance to dominate apple aroma in ciders with the addition of pear juice [2]. The aim of the study was to investigate the composition of volatile compounds in ciders produced from three varieties of regional apples and their mixtures with pear juice during fermentation at two different temperatures.

## 2. Results

### 2.1. Volatile Compounds in Apple and Apple/Pear Juices

In this study, volatile compositions of apple juice and apple juice and pear juice mixes from three apple cultivars and one pear cultivar were assessed by means of gas chromatography coupled with mass spectrometry. In the apple juices, 43 volatile compounds were identified (Table 1) and in the pear juice/apple juice mixes, 46 volatile components could be recognized. They were divided into five chemical groups. The largest number of identified compounds in apple juices were esters (27 compounds) and the second largest group were alcohols (10 compounds). Other chemical groups consisted of aldehydes (three compounds), sesquiterpenes (two compounds) and acids (and compound). Pear and apple juice mixes also contained five different chemical groups of components, but 30 esters instead of 27 were identified. *Idared* and *Konferencja* juices mixture (IK) had the highest relative abundance of esters out of the analyzed juices (84.37% of peaks area). The addition of pear increased contributions of esters in the aroma profile of this juice, because esters constituted 80.92% of peak area of *Idared* juice (I). The lowest relative concentration of esters, which equaled 28.63%, was identified in the *Cortland* and *Konferencja* juice mixture (CK) and was similar to the relative concentration of esters in *Cortland* juice (C)—29.94%. In *Gala* variety juice (G) and *Gala* and *Konferencja* juices (GK) mixture, esters constituted, respectively, 65.54% and 66.43%. Second largest chemical group of compounds which were identified in juices was alcohols. Alcohols constituted the greatest share of volatiles in C (60.57%) and CK (65.17%), over two times greater than esters in these juices. The lowest abundance of alcohols was found in IK (9.71%) and the next in GK (13.38%). The same apple juices, without the contribution of pear juice, were characterized by greater amounts of alcohols: 14.35% in I and 23.22% in G. A greatest amount of aldehydes was found out in GK (10.35%), and in the rest of the samples aldehydes constituted only a minuscule amount of volatile components (0.28–1.37%). Addition of pear juice to each apple juice resulted in a lower relative share of sesquiterpenes in the composition of volatiles. Sesquiterpenes constituted the largest part of volatile composition in C (4.37%) and CK (3.28%) compared to the other samples, wherein the contribution of sesquiterpenes to aroma profile was lower, from 1.96% in I to 1.07% in GK. Acids, with only one compound in this group, were an important part of the aromas of G and GK, wherein they constituted, respectively, 9.28% and 8.59% of total volatiles. In other juices, the amount of acids was 2–4 times lower than in the juices with higher concentrations (2.41–4.3%). For the I and IK, the main aroma-forming compounds were ethyl 2-methylbutanoate, 2-methylpropyl acetate and hexyl acetate, which are associated with sweet, appley and banana aromas [12]. G and GK were also characterized by large concentrations of hexyl acetate in the overall aromatic profile. Main components of the C and CK were not only esters, such as 2-methyl propyl acetate acetate and butyl hexanoate, but alcohols such as (*Z*)-hex-2-en-1-ol and (*E*)-hex-2-en-1-ol. Three compounds, characterized by pear aroma, were not present in the samples without pear addition, but were identified in all samples with the addition of pear juice [13]. These were three esters: ethyl (*Z*)-dece-4-noate, methyl (2*E*,4*Z*)-deca-2,4-dienoate, ethyl (2*E*,4*Z*)-deca-2,4-dienoate.

### 2.2. Volatile Compounds in Ciders

Gas chromatography and mass spectrometry allowed for the identification of 32 volatile compounds in ciders (Table 2). As in the case of juices, volatile components identified in ciders could be divided into five different chemical groups, including esters (21 compounds), alcohols (four compounds), acids (four compounds), sesquiterpenes (two compounds) and aldehydes (one compound). Esters had the greatest contribution to the volatile composition in the case of every sample. The lowest relative abundance of esters was found in the case of the *Gala* variety cider fermented at 20 °C (G20), where they contributed to 58.81% total volatiles. The highest contribution of esters was found for the *Cortland* variety cider fermented at 15 °C (C15) and it equaled 79.58%. Ciders, fermented at 15 °C and 20 °C prepared with *Konferencja* variety juice, were characterized by higher contents of esters in the cases of apple varieties *Idared* and *Gala* (IK15, IK20, GK15, GK20) compared to the *Idared* and *Gala* ciders without pear addition (I15, I20, G15, G20). A different occurrence can be seen in the case of the *Cortland* variety cider. Ciders prepared from *Cortland* variety apple juice, fermented at 15 °C and 20 °C (C15, C20), are characterized by higher relative concentrations of esters than *Cortland/Konferencja* ciders (CK15, CK20). The alcohols constituted not only the second biggest group of compounds by the number of compounds identified, but also by their total relative abundance. The highest contribution of alcohols to the aroma profiles of ciders was found for G20, wherein they constituted 36.71% of total volatiles. The lowest contribution of alcohols to the aroma profile (16.07%) was found for C15. Addition of pear juice increased the amount of alcohols in *Cortland* variety ciders from 18.94% in C15 to 25.76% in CK15 and from 20.53% in C20 to 32.19% in CK20. In the cases of *Gala* and *Idared* cultivars, addition of pear juice had the opposite effect. Relative concentrations of alcohols decreased, from 24.6% for I15 to 19.9% for IK15; and from 22.45% for G15 to 19.43% for GK15. A similar trend could be noted for ciders fermented at 20 °C. The proportion of alcohols regarding volatile composition in I20 was equal to 29.04% and was smaller in IK20: 27.65%; in G20 alcohols composed 36.7% of volatiles, and in GK20 28.97%. Alcohol (except in GK20) was phenylethyl alcohol, which is produced by yeast and has a bread-like and flowery aroma [14]. Acids composed the third largest group of compounds and relative concentrations of these chemicals ranged from 3.16% in C15 to 9.18% in I15. A similar trend as in the case of alcohols could be seen in the ciders produced from mixed pear/apple juice. I15 and I20 were characterized by higher amounts of acids (9.18% and 8.29%, respectively) than IK15 and IK20 (4.27% and 6.36%); G15 and G20 also contained more acids in the volatile composition (4.7% and 4.15% respectively) than GK15 and GK20, which contained 3.28% and 3.67% of acids. Most prominent acid in all the ciders was octanoic acid, characterized by oily and waxy aroma [15]. Only one aldehyde, (*E*)-hex-2-enal, was found in the volatile compositions of ciders. It is a compound which has leafy aroma [16]. Its presence was not detected in four of the tested ciders (IK15, I20, C20, G20). (*E*)-hex-2-enal was found only in small quantities in other samples and constituted from 0.16% of volatiles in I15 to 0.96% in GK15. Its concentration was lower for the ciders prepared with pear juice addition only in the cases of IK15 and CK15. Sesquiterpenes were present in minuscule amounts in all tested ciders and their concentrations ranged from the lowest in GK20 (0.25% of all volatiles) to the highest in IK20 (0.83%) of all volatiles.

### 2.3. Carbohydrate and Ethanol Concentrations and Degree of Attenuation

The concentration of carbohydrates was determined in the juices prior to the fermentation by the means of high performance liquid chromatography (HPLC). Carbohydrate concentration has been presented as a sum of fermentable sugars (glucose, fructose and sucrose) (Table 3 and Table 4). The highest concentration of sugars in the juices was found for GK (13.9% *w/w*) and the lowest for I (10.9% w/w). At the end of fermentation, 14 days after inoculation with yeast, concentration of sugars was significantly lower and ranged from 2.23% (*w/w*) for I20 to 4.38% (*w/w*) for C15. The highest alcohol concentration was assessed in GC20 and was equal to 6.79% (*v/v)* and the lowest was determined in C15: 4.12% (*v/v*). The highest degree of fermentation was achieved by I20 and was equal 86.7%, and the lowest was achieved by C15, which equaled 60.54%.

## 3. Discussion

### 3.1. Volatile Compounds in Apple and Apple/Pear Juices

The aroma of apple juice has been the subject of much research and discussion [24,25,26]. Depending on the apple variety, climate and the use of different cultivation techniques, the researchers have pointed out significant differences between the aromatic profiles of analyzed juices. It has also been shown that differences in the production technology can have significant impacts on the aroma profiles of apple juices [27,28,29]. In this study, an attempt was made to show the differences in the quality of apple juice, which differ not only by the apple variety, such as *Gala*, *Cortland* and *Idared*, but to also assess how addition of pear *Konferencja* juice, can affect the volatile profile. Current knowledge about the formation of cider aromas resulting from mixtures of apple-pear juices is not sufficient and does not include a detailed analysis of the changes occurring in the volatile components of the juice building the aroma of cider which is formed during the fermentation process. Most researchers indicate methyl and ethyl esters of carboxylic acids as the main groups of compounds building the aroma of apple and pear juice [13,29,30,31,32,33]. In these studies, out of 46 marked chemical compounds which composed the aromas of apple and apple-pear juices, 32 compounds belonged to the group of esters. The main esters in the studies by Kato et al. [34], Aprea et al. [35] and Gasperi et al. [36] were: hexyl acetate, hexan-1-ol, butyl acetate and (*Z*)-hex-3-enyl acetate. In the tests performed, a large share of hexyl acetate (23–29%) was found as a compound shaping the aromatic profile of apple juice from the *Idared* and *Gala* varieties and their mixtures with pears (GK, IK). Other researchers, such as Xiaobo and Jiewen [37], who studied exotic Asian apples such as *Fuji*, *Jina* and *Huaniu*, also indicated the presence of the above-mentioned esters and a significant shares of, among others, propyl butanoate and propyl acetate. Other important groups of chemical compounds found in apple juices, which have been indicated by many studies, are alcohols ((*E*)-hex-2-en-1-ol, (*Z*)-hex-2-en-1-ol (*Z*), 2-methyl butan-1-ol, (*Z*)-hex-4-enol) and aldehydes ((*E*)-hex-2-enal, hexenal, nonanal, dodecanal) [29,32,33,34,35,36,37]. The research also showed significant influences of alcohols and aldehydes, such as (*E*)-hex-2-en-1-ol and (*Z*)-hex-2-en-1-ol, which dominated mainly in the juice of the *Cortland* variety, in building the aroma profiles of the analyzed juices. Other compound, belonging to the sesquiterpene group ((*E*, *E*)-α-farnesene) was found in the analyzed juices at a level close to the values presented by other researchers [38]. A relationship was also found between the content of farnesene isomers and the apple variety from which the juice originated. Ahn et al. [39] pointed to Cortland as the richest in the α-farnesene variety, placing apples of the Idared and Gala varieties behind it. Similar relations between the participation of farnesene in juices were also confirmed in this study. The addition of pear juice slightly decreased the share of farnesene in the overall aromatic profiles of the analyzed juices. Many researchers have shown that the aroma of pear juice is greatly dependent on the varietal factors, climate characteristics, cultivation techniques and degree of ripeness of the fruit used [13,40]. The main compounds characterized in the aromatic profiles of pears have included ethyl acetate, hexanal, ethyl butyrate and ethyl hexanoate. The addition of pear juice to apple juice affected the aromatic profile of the finished product. The main differences in the aromas of ciders are probably due to compounds such as ethyl-(2*E*,4*Z*)-deca-2,4-dienoate, which has a characteristic pear smell and is referred to by some researchers as a “pear ester” [13,41]. In the samples of mixed apple and pear juices, the percentage of this compound was significantly higher than in apple juices.

### 3.2. Volatile Compounds in Ciders

The amounts of volatile compounds contained in the tested cider samples depend on many variables. In addition to the varietal factor, fermentation temperature and types of microorganisms conducting the fermentation process, the analytical method used also plays a significant role in the amounts of the determined compounds (the main ones include GC, GC-MS, GC-MS/MS, GC-O, GC-MS-O) [10,42,43]. In this work, the most represented compounds identified in ciders included methyl and ethyl esters of carboxylic acids. The most important for building the aromatic profile were: ethyl hexanoate, ethyl acetate, 2-phenylethyl acetate, ethyl decanoate and ethyl dodecanoate. These compounds belong to the basic esters that build the aromas of apple and pear juices and are formed during fermentation processes with the use of *Saccharomyces cerevisiae* yeast [42,44,45]. Gamero et al., analyzing the effects of temperatures in the range 12–28 °C on the production of aromatic compounds by *S. cerevisiae*, used synthetic substrates to eliminate the effect of the matrix on the by-products of alcoholic fermentation [46]. During the research, it was found that at elevated fermentation temperature, *S. cereviasie* yeast produces increased amounts of esters such as ethyl acetate, 2-methylpropyl acetate, 3-methylbutyl acetate, hexyl acetate, ethyl hexanoate, ethyl octane and ethyl decanoate. Rolerro et al., while examining the effects of fermentation temperature and substrate supplementation on the shaping of the aromatic profiles of grape wines, found that the production of esters such as ethyl hexanoate or ethyl octanoate depend not only on the fermentation temperature, but also on the proportion of free nitrogen and fatty acids in the substrate [47]. In this work, a significant influence of fermentation temperature on the percentage share of esters in the aromatic profiles of the studied ciders was also observed. Fermentation temperature played a key role in the case of compounds such as ethyl hexanoate, ethyl octanoate and ethyl dodecanoate, whose percentages were higher in ciders fermented at 15 °C. Differences in observed relationships between fermentation temperature and the share of esters in the aromatic profiles of the analyzed ciders were due to differences between fermentation settings (contents of microelements, acidity or contents of extract). Ciders and cider musts are characterized by a much lower dry matter concentration, density, acidity and nitrogen content than wine musts [48]. Other important groups of compounds responsible for the cider aroma are alcohols and carboxylic acids. The research showed that the most important thing for shaping the aromatic profiles of the tested ciders from the *Cortland*, *Idared* and *Gala* juices were alcohols such as 3-methyl butan-1-ol, 2-methyl butan-1-ol, hexan-1-ol and phenylethyl alcohol, and acids such as octanoic and decanoic. Lobo et al., while analyzing Spanish ciders from Asturias and the Basque country have found alcohols such as butan-2-ol, propan-1-ol and hexan-1-ol, and acids such as hexanoic, octanoic and decanoic as dominant volatile compounds. The contents of these compounds in the cider production process increased significantly, which corresponds with the results obtained in this study [49]. Anton et al., analyzing the aromatic profiles of ciders using the yeast of the *S. cerevisiae* species and wild yeast (spontaneous fermentation) showed that the dominant alcohols in both cases were 2-methyl-butan-1-ol, 1-propan-1-ol, iso-butan-1-ol and 3-methylbutylalcohol and acids such as hexanoic, decanoic and octanoic [50]. Other researchers also point out important role of such volatiles as phenylethyl alcohol, whose aroma is described as honey-like and floral; hexyl alcohol, characterized by grassy notes; and alcohols such as 2-methyl-butan-1-ol and 3-methyl-butan-1-ol [51,52]. Sesquiterpenes are the group of compounds of great importance for the cider aroma, constituting 0.6% of all identified compounds. In this study, the content of compounds such as ((*Z*,*E*)-α-Farnesene, (*E*,*E*)-α-Farnesene in all tested cider musts) was determined. α-Farnesene isomers are often marked as volatile components in both apple and pear musts and ciders [38,53,54].

Current research in the field of cider production technology mainly concentrates on the contents of polyphenolic compounds and the analysis of aromatic profiles. These compounds play an important role due to their participation in the creation of the desired organoleptic characteristics and the bioactive and health-promoting properties of ciders. The current state of knowledge is also being expanded by research about issues related to the production of ciders using malo-lactic fermentation, used to obtain ciders with reduced acidity [55,56,57].

## 4. Materials and Methods

### 4.1. Raw Material

Juices from three apple cultivars: *Idared*, *Cortland* and *Gala* and pear cultivar: *Konferencja* were obtained from local orchard in Lubuskie voivodeship in village Solniki. Juices were pasteurized on PA1500 Pasteurizer (Voran Company, Pichl bei Wels, Austria) by juice producer “Słoneczny Sad” (Solniki, Poland) under the following conditions: 74 °C for 15 s under normal pressure.

### 4.2. Biological Material

Dry yeast used in this study to ferment apple and apple-pear musts to acquire cider was *Saccharomyces cerevisiae* Safdistill C-70 strain. It was obtained from the Fermentis division of Lesaffre company (Marqen Baroeul, France). Prior to inoculation, 2.5 g (dry weight) of dry yeast was rehydrated in 20 mL of sterile, distilled water and thermostated at 30 °C for 30 min with regular agitation.

### 4.3. Cider Production

Fermentation of apple juices and apple juice/pear juice mixes was carried out in 5.5 L glass vessels in a single repetition; 5 L of juice was inoculated by 20 mL of yeast solution. The fermentation was carried out for 14 days. Six cider variants were fermented at 15 °C: 100% *Idared* apple juice (I15); 100% *Cortland* apple juice (C15); 100% *Gala* apple juice (G15); 80% *Idared* apple juice and 20% *Konferencja* pear juice mix (IK15); 80% *Cortland* apple juice and 20% *Konferencja* pear juice mix (IK15); 80% *Gala* apple juice and 20% *Konferencja* pear juice mix (GK15). Six cider variants were fermented at 20°C: 100% *Idared* apple juice (I20); 100% *Cortland* apple juice (C20); 100% *Gala* apple juice (G20); 80% *Idared* apple juice and 20% *Konferencja* pear juice mix (IK20); 80% *Cortland* apple juice and 20% *Konferencja* pear juice mix (IK20); 80% *Gala* apple juice and 20% *Konferencja* pear juice mix (GK20). Ciders, after fermentation, were bottled and kept at 4 °C.

### 4.4. Volatile Compounds Analysis

Solid-phase microextraction (SPME) techniques were applied for the isolation and concentration of volatile flavor compounds [58]. A representative cider sample (40 mL) was transferred to a 100 mL septum-closed vials with 0.5 µg 2-undecanone added as an internal standard for quantification. Samples were held in an 80 °C water bath and shaken during the isolation. Each sample was allowed to equilibrate and absorb onto the SPME fiber (2 cm–50/30 µm DVB/CAR/PDMS; Supelco, Inc., Bellefonte, PA, USA) for 45 min. A gas chromatograph Clarus 680 coupled with a mass spectrometry detector (GC-MS) SQ 8S by Perkin Elmer, with a column ELITE-5MS (Crossbond 5% diphenyl and 95% dimethylpolysiloxane) 30 m × 0.25 mm ID × 0.25 μm film. Scanning was performed from 50 to 300 m/z in electron impact (EI) at 70 eV, mode at 5 scan s^−1^. Analyses were carried out using helium as carrier gas at a flow rate of 1.0 mL min^−1^ in a split ratio 1:10 and the following temperature program: (a) 40 °C for 3 min; (b) rate of 5.0 °C min^−1^ from 40 to 160 °C; (c) rate of 30 °C min^−1^ from 160 to 280 °C. Injector was held constantly at 220 °C. Compounds were identified by using 3 different analytical methods: (1) retention indices (KI), (2) GC-MS retention times (authentic chemicals) and (3) comparison of mass spectra, with database (NIST 11 Mass Spectral and Retention Index Libraries (The National Institute of Standards and Technology, Gaithersburg, MD, USA) with similarity indexes set above 90%. For comparison of obtained mass spectra Automated Mass Spectral Deconvolution and Identification System (AMDIS) software version 2.73 was used.

Linear retention indices were calculated against C6-C30 *n*-alkanes mix (Sigma-Aldrich, Saint Louis, MO, USA).

Quantification was based on the peak area normalization. For data processing, ACD/Spectrus Processor 2017.2.1 software (Advanced Chemistry Development, Inc. Toronto, Ontario M5C 1B5, Canada) was used.

### 4.5. Carbohydrates and Ethanol Concentration and Degree of Attenuation

Concentration of carbohydrates (sucrose, glucose and fructose) in ciders, apple and pear juices and ethanol content of ciders was assessed by the means of high performance liquid chromatography (HPLC). Samples of cider and juice were 3-fold diluted with de-ionized water and filtered through 0.2 µm nylon membrane filter. Analyses were performed using Shimadzu Prominence chromatograph (Shimadzu Corp., Tokyo, Japan) equipped with Rezex ROA-Organic Acid H+ column (300 × 7.8 mm) (Phenomenex, Torrance, USA). The samples were loaded into 20 µL injection loop and eluted at 50 °C with 0.005 M H_2_SO_4_ as a mobile phase at a flow rate of 0.4 mL min^−1^. The compounds were detected using Shimadzu RID-10A refractive index detector; detection temperature was maintained at 50 °C. Integration and quantification of chromatograms were performed using Chromax 10 software (Pol-Lab, Warsaw, Poland). Degree of attenuation was calculated using the formula below:

(SJ − SC)/SJ = Degree of attenuation

SJ-carbohydrate content in juice

SC-carbohydrate content in finished cider

### 4.6. Statistical Analysis

The results of volatile compounds concentration analysis and sugars and ethanol content were statistically analyzed using Duncan’s test at the significance level of α = 0.05. In the volatile analysis, three-way analysis of variance (ANOVA) was performed to detect significant differences in the analyte concentrations depending on the main factors studied (fermentation temperature, cultivar of apples and addition of pear juice). Statistica software in version 10 was used for data processing.

## 5. Conclusions

The sensory qualities of cider are linked to the volatile compounds—which are composed of volatiles present in the raw material used for cider-making—and to the metabolites created by the microorganisms during the fermentation process. In this study, 46 volatiles have been identified in the pear juice/apple juice mixes and 33 volatiles have been found in the ciders prepared from these juices. The main chemical compounds found in *Idared* apple juice were 2-methyl ethyl butanoate, hexyl acetate and 2-methylpropyl acetate. The main aromatic constituents in the volatilome of *Cortland* apple variety juice were (*E*)-hex-2-en-1-ol, 2-methylpropyl acetate and (*Z*)-hex-2-en-1-ol (*Z*). In the composition of volatile profile of *Gala* juice, (*E*)-hex-2-enol, hexyl acetate and ethyl oct-2-enoate were recognized as the most abundant constituents. The addition of pear juice added esters with a characteristic pear aroma, such as methyl (2*E*,4*Z*)-deca-2,4-dienoate and ethyl (2*E*, 4*Z*)-deca-2,4-dienoate. Fermentation of juices changed composition of the juices’ volatilomes. In the ciders, the main chemical components were esters. The most abundant esters determined in all of the ciders were ethyl decanoate and ethyl octanoate. In the ciders prepared from juice with pear juice addition, ethyl (2*E*,4*Z*)-deca-4-dienoate, characteristic of the pear aroma, was found. Ciders were characterized by a higher degree of alcohols, such as hexan-1-ol, phenylethyl alcohol and 2-methyl butan-1-ol, in the volatile profiles than in juices from which they were prepared. Ciders were also characterized by lower relative abundances of sesquiterpenes, such as (*Z*,*E*)-α-farnesene and (*E*,*E*)-α-farnesene, in the volatilome than in apple and pear juices.

## Figures and Tables

**Table 1:** Volatile compounds identified in analyzed juices.

Compound	Aroma Descriptor ^1^	Retention Indices		Chemical Family	I	IK	C	CK	G	GK ^2^
		Exp.	Lit.		[%] of Total Peak Area					
Butan-1-ol	fruity, medicinal	674	675	Alcohols	0.72 ± 0.22b	0.01 ± 0.01c	0.07 ± 0.0c	0.01 ± 0.01c	2.31 ± 1.15a	0.09 ± 0.05c
Propyl acetate	sweet, fruity	705	708	Esters	0.01 ± 0.01d	0.59 ± 0.23a	0.06 ± 0.04bc	0.08 ± 0.05bc	0.10 ± 0.06b	0.02 ± 0.02d
2-methylbutan-1-ol	winey, oniony,	740	745	Alcohols	3.97 ± 1.98a	3.09 ± 1.84b	2.28 ± 1.04c	2.34 ± 1.23c	0.80 ± 0.53d	0.36 ± 0.24e
Butyl acetate	appley	812	812	Esters	0.68 ± 0.44a	0.49 ± 0.32b	0.05 ± 0.04d	0.01 ± 0.01d	0.15 ± 0.08c	0.05 ± 0.03d
(*E*)-hex-2-en-1-ol	leafy, fresh	832	840	Alcohols	0.42 ± 0.28d	0.38 ± 0.21d	13.00 ± 5.42b	18.30 ± 6.08a	3.98 ± 2.15c	3.42 ± 1.78c
Propan-2-yl butanoate	banana-like	843	843	Esters	0.10 ± 0.06b	0.05 ± 0.03c	0.45 ± 0.31a	0.16 ± 0.09b	0.11 ± 0.06b	0.07 ± 0.04c
Ethyl 2-methylbutanoate	appley	846	845	Esters	19.50 ± 4.38a	19.00 ± 5.02a	1.62 ± 0.76d	2.43 ± 1.15c	5.62 ± 2.44b	5.67 ± 2.83b
(*E*)-hex-2-enal	leafy	849	847	Aldehydes	0.01 ± 0.01d	1.30 ± 0.95b	0.37 ± 0.24c	0.05 ± 0.03d	0.07 ± 0.04d	10.10 ± 4.32a
(*E*)-hex-2-en-1-ol	leafy, winey, appley	855	854	Alcohols	1.70 ± 1.05d	1.04 ± 0.82d	43.10 ± 8.53a	43.40 ± 9.12a	10.20 ± 6.41b	6.63 ± 2.81c
Hexan-1-ol	flowery	865	863	Alcohols	1.26 ± 0.74a	0.01 ± 0.01d	0.70 ± 0.53b	0.01 ± 0.01d	0.20 ± 0.13c	0.01 ± 0.01d
(*Z*)-hex-4-en-1-ol	fruity, fresh,	873	870	Alcohols	5.40 ± 3.11a	4.17 ± 2.83b	0.01 ± 0.01d	0.06 ± 0.04d	4.64 ± 2.72ab	2.39 ± 1.42c
2-methylpropyl acetate	sweet, banana-like	883	883	Esters	25.10 ± 9.42a	25.60 ± 10.83a	6.47 ± 4.18bc	5.53 ± 2.78d	7.64 ± 4.72b	6.18 ± 2.81cd
Propyl butanoate	fruity,	904	898	Esters	0.01 ± 0.01e	0.20 ± 0.12bc	0.09 ± 0.05d	0.33 ± 0.18a	0.16 ± 0.09c	0.13 ± 0.07c
Butyl propanoate	earthy, sweet	916	915	Esters	0.34 ± 0.21e	0.65 ± 0.29d	1.72 ± 0.94ab	1.56 ± 0.82b	1.75 ± 0.97a	1.23 ± 0.72bc
Pentyl acetate	banana-like, etheric. fruity	923	920	Esters	0.92 ± 0.42c	1.99 ± 1.05a	0.55 ± 0.34d	0.14 ± 0.08e	0.85 ± 0.42c	1.68 ± 1.12b
Tert-butyl butanoate	fruity, sweet, appley	955	956	Esters	0.14 ± 0.08c	0.13 ± 0.09c	1.18 ± 0.66a	1.10 ± 0.54a	0.19 ± 0.12bc	0.13 ± 0.08c
2-methylbutyl propanoate	fruity, etheric	963	965	Esters	0.03 ± 0.02c	0.28 ± 0.16a	0.06 ± 0.04b	0.06 ± 0.05b	0.02 ± 0.02c	0.03 ± 0.02c
Benzaldehyde	oleic, nutty, sour cherry	970	960	Aldehydes	0.03 ± 0.02d	0.07 ± 0.05d	0.35 ± 0.18a	0.15 ± 0.09c	0.15 ± 0.11c	0.22 ± 0.14bc
Heptan-1-ol	leafy, musty	983	980	Alcohols	0.10 ± 0.06bc	0.25 ± 0.16a	0.31 ± 0.23a	0.06 ± 0.04c	0.05 ± 0.04c	0.01 ± 0.01c
Butyl butanoate	sweet, fruity, fresh	1001	998	Esters	0.60 ± 0.27c	1.98 ± 0.82a	0.11 ± 0.06d	1.33 ± 0.78b	1.16 ± 0.72b	1.76 ± 1.02a
hex-5-enyl acetate	sweet, leafy, fresh	1011	1010	Esters	0.10 ± 0.06b	0.08 ± 0.05b	0.09 ± 0.05b	0.15 ± 0.09a	0.15 ± 0.06a	0.17 ± 0.08a
Hexyl acetate	sweet, green apple, banana	1022	1022	Esters	29.80 ± 6.72a	29.80 ± 8.44a	5.55 ± 2.31c	5.30 ± 2.12c	23.30 ± 7.12b	28.60 ± 5.15a
Ethyl hex-2-enoate	sweet, fresh, leafy,	1038	1040	Esters	0.35 ± 0.18a	0.06 ± 0.04c	0.07 ± 0.03c	0.13 ± 0.06b	0.05 ± 0.04c	0.10 ± 0.07bc
Butyl 2-methylbutanoate	fruity, coconutty,	1050	1045	Esters	0.68 ± 0.32c	0.58 ± 0.27c	0.37 ± 0.14d	0.30 ± 0.16d	2.56 ± 1.82a	2.02 ± 1.04b
Butyl 3-methylbutanoate	apple skin	1067	1070	Esters	0.05 ± 0.03d	0.28 ± 0.12ab	0.02 ± 0.02d	0.23 ± 0.11b	0.15 ± 0.08c	0.12 ± 0.07c
Octan-1-ol	citrusy, flowery, aldehydy	1082	1080	Alcohols	0.42 ± 0.28c	0.32 ± 0.20d	0.84 ± 0.44ab	0.80 ± 0.38b	0.91 ± 0.47a	0.42 ± 0.31c
Pentyl butanoate	pineappley, banana, tropical	1099	1098	Esters	0.14 ± 0.09c	0.15 ± 0.06c	0.97 ± 0.58a	0.73 ± 0.42b	0.17 ± 0.12c	0.20 ± 0.14c
Hexyl propanoate	fruity, green pear	1111	1114	Esters	0.05 ± 0.03e	0.02 ± 0.02e	0.40 ± 0.28c	0.16 ± 0.12d	2.03 ± 1.05a	1.31 ± 0.72b
Heptyl acetate	lemongrass	1118	1118	Esters	0.05 ± 0.04d	0.06 ± 0.04d	0.45 ± 0.22a	0.30 ± 0.18b	0.10 ± 0.08c	0.11 ± 0.07c
2-methylpentyl butanoate	appley, fruity	1145	1150	Esters	0.14 ± 0.08a	0.12 ± 0.08a	0.14 ± 0.06a	0.11 ± 0.07ab	0.09 ± 0.05b	0.15 ± 0.12a
2-methylhexyl propanoate	fruity, appley, sweet	1154	1155	Esters	0.59 ± 0.33a	0.38 ± 0.23b	0.16 ± 0.07cd	0.20 ± 0.14c	0.10 ± 0.06d	0.09 ± 0.06d
Octan-1-ol	woody, herbal, fresh	1174	1180	Alcohols	0.31 ± 0.22b	0.41 ± 0.28a	0.07 ± 0.04d	0.11 ± 0.06cd	0.04 ± 0.03e	0.01 ± 0.01e
Butyl hexanoate	pineappley, waxy, fruity	1196	1198	Esters	0.30 ± 0.18d	0.29 ± 0.11d	8.86 ± 3.12a	6.62 ± 2.83bc	5.30 ± 2.41c	5.25 ± 2.62c
Ethyl (*Z*)-oct-2-enoate	tropical, waxy, fruity,	1200	1202	Esters	0.36 ± 0.28c	0.45 ± 0.31c	0.19 ± 0.08d	0.44 ± 0.28c	12.30 ± 2.34a	10.50 ± 3.12b
Octyl acetate	mushroom-like, herbal, earthy	1216	1212	Esters	0.65 ± 0.36a	0.13 ± 0.07b	0.07 ± 0.05c	0.09 ± 0.06bc	0.10 ± 0.06b	0.11 ± 0.08b
2,4-dimethylobenzaldehyde	chemical, vanilla, sour cherry,	1222	1230	Aldehydes	0.31 ± 0.18a	0.02 ± 0.02d	0.06 ± 0.05cd	0.08 ± 0.04c	0.15 ± 0.10b	0.03 ± 0.02d
Decanoic acid	appley, fruity, banana	1242	1245	Acids	2.41 ± 1.63e	2.83 ± 1.82d	4.30 ± 2.93c	2.64 ± 1.44de	9.28 ± 2.55a	8.59 ± 3.12b
3-methylbutyl hexanoate	pineappley, fruity	1258	1259	Esters	0.11 ± 0.08bc	0.05 ± 0.04d	0.01 ± 0.01d	0.08 ± 0.02c	0.07 ± 0.04cd	0.46 ± 0.28a
(*Z*)-dec-3-en-1-ol	waxy, fruity	1266	1275	Alcohols	0.05 ± 0.04c	0.03 ± 0.02c	0.19 ± 0.12a	0.08 ± 0.06b	0.09 ± 0.05b	0.04 ± 0.03c
Heptyl 2-methylbutanoate	appley	1339	1342	Esters	0.06 ± 0.04c	0.06 ± 0.03c	0.16 ± 0.12a	0.10 ± 0.04b	0.09 ± 0.05b	0.02 ± 0.02d
Ethyl (*Z*)-dec-4-enoate	grapefruity, waxy, fruity, appley	1361	1364	Esters	0.00b	0.01 ± 0.01a	0.00b	0.01 ± 0.01a	0.00b	0.01 ± 0.01a
Methyl (2*E*,4*Z*)-deca-2,4-dienoate	pear	1386	1383	Esters	0.00b	0.02 ± 0.01a	0.00b	0.04 ± 0.02a	0.00b	0.03 ± 0.02a
Hexyl hexanoate	tropical, waxy, fruity	1389	1385	Esters	0.06 ± 0.04d	0.78 ± 0.42b	0.07 ± 0.04d	0.80 ± 0.38b	1.23 ± 0.67a	0.15 ± 0.11c
Ethyl (2*E*,4*Z*)-deca-2,4-dienoate	tropical, pear	1479	1474	Esters	0.00b	0.09 ± 0.06a	0.00b	0.11 ± 0.07a	0.00b	0.08 ± 0.05a
(*Z,E*)-α-Farnesene	woody	1491	1495	Sesquiterpenes	0.23 ± 0.11b	0.21 ± 0.12b	0.31 ± 0.18a	0.33 ± 0.19a	0.11 ± 0.08c	0.09 ± 0.07c
(*E,E*)-α-Farnesene	woody, citrusy	1507	1505	Sesquiterpenes	1.73 ± 0.98c	1.48 ± 0.82d	4.06 ± 2.02a	2.95 ± 1.62b	1.49 ± 1.12d	0.98 ± 0.72e

^1^ [17,18,19,20,21,22,23]; ^2^ I—*Idared* apple variety juice; IK—*Idared* apple (80%) and *Konferencja* pear (20%) juices mix; C—*Cortland* apple variety juice; CK-+—*Cortland* apple (80%) and *Konferencja* pear (20%) juices mix; G—*Gala* apple variety juice; GK—*Idared* apple (80%) and *Konferencja* pear (20%) juices mix. Values are expressed as means (n = 2) ± standard deviations. Mean values with different letters (a, b, c, d, e) within the same column are statistically different (*p*-value < 0.05).

**Table 2:** Volatile compounds identified in analyzed ciders.

Compound	Aroma Descriptor ^1^	Retention Indices		Chemical Family	I15	IK15	C15	CK15	G15	GK15	I20	IK20	C20	CK20	G20	GK20 ^2^
		Exp.	Lit.		[%] of Total Peak Area											
3-methylbutan-1-ol	fusel, alcoholic	699	705	Alcohols	6.49 ± 1.98c	4.19 ± 1.42e	7.11 ± 2.77bc	8.71 ± 2.34b	9.98 ± 2.18a	7.96 ± 3.02b	5.36 ± 1.04d	4.67 ± 1.88e	4.16 ± 1.27e	4.96 ± 2.21de	9.09 ± 2.66ab	7.82 ± 2.68b
2-methylbutan-1-ol	winey, oniony	740	745	Alcohols	2.95 ± 1.42c	4.68 ± 2.12a	1.09 ± 0.68e	3.51 ± 1.91b	2.32 ± 0.98d	0.93 ± 0.44ef	4.49 ± 1.98ab	2.72 ± 1.28c	0.68 ± 0.34f	3.17 ± 1.86c	3.88 ± 1.72b	0.40 ± 0.21g
Ethyl butanoate	fruity	805	794	Esters	0.29 ± 0.17b	0.05 ± 0.03e	0.18 ± 0.09c	0.16 ± 0.07c	0.10 ± 0.06de	0.21 ± 0.11bc	0.44 ± 0.28a	0.00f	0.00f	0.05 ± 0.03e	0.00f	0.06 ± 0.04e
Butyl acetate	appley	812	812	Esters	1.27 ± 0.48bc	0.00g	0.22 ± 0.14e	1.71 ± 0.88ab	0.05 ± 0.03f	0.48 ± 0.32d	1.89 ± 0.92a	0.00g	0.00g	1.51 ± 0.74b	0.00g	0.19 ± 0.08e
(*E*)-hex-2-enal	leafy	849	847	Aldehydes	0.16 ± 0.09d	0.00e	0.73 ± 0.31b	0.36 ± 0.14c	0.91 ± 0.68a	0.96 ± 0.56a	0.00e	0.44 ± 0.23c	0.00e	0.67 ± 0.28b	0.00e	0.74 ± 0.43b
Hexan-1-ol	flowery	865	863	Alcohols	0.6 ± 0.29e	1.15 ± 0.72d	2.31 ± 0.84c	0.79 ± 0.44e	0.97 ± 0.63de	2.13 ± 1.01c	0.62 ± 0.38e	9.86 ± 2.92a	4.27 ± 1.34b	3.79 ± 1.45b	10.59 ± 3.18a	10.57 ± 3.24a
2-methylbutyl acetate	banana	883	883	Esters	4.44 ± 2.72a	0.07 ± 0.04f	0.00g	4.76 ± 2.56a	0.00g	0.13 ± 0.10	1.70 ± 0.92c	1.09 ± 0.63d	1.86 ± 1.32c	3.32 ± 1.78b	0.63 ± 0.34e	0.46 ± 0.22e
Ethyl pentanoate	sweet	903	901	Esters	0.02 ± 0.01a	0.01 ± 0.01a	0.01 ± 0.01a	0.02 ± 0.01a	0.01 ± 0.01a	0.00 ± 0.01a	0.01 ± 0.01a	0.01 ± 0.01a	0.01 ± 0.01a	0.01 ± 0.01a	0.01 ± 0.01a	0.01 ± 0.01a
Butyl propanoate	earthy	916	915	Esters	0.01 ± 0.01b	0.01 ± 0.01b	0.01 ± 0.01b	0.02 ± 0.01b	0.01 ± 0.01b	0.04 ± 0.02a	0.01 ± 0.01b	0.01 ± 0.01b	0.01 ± 0.01b	0.01± 0.01b	0.01 ± 0.01b	0.01 ± 0.01b
Pentyl acetate	etheric, fruity, banana	923	920	Esters	0.06 ± 0.04a	0.01 ± 0.01b	0.01 ± 0.01b	0.08± 0.06a	0.00b	0.02± 0.01b	0.01± 0.01b	0.01± 0.01b	0.00b	0.00b	0.00b	0.01± 0.01b
Tert-butyl butanoate	fruity, appley	955	956	Esters	0.01 ± 0.01a	0.01 ± 0.01a	0.01 ± 0.01a	0.01 ± 0.01a	0.01 ± 0.01a	0.01 ± 0.01a	0.01 ± 0.01a	0.01 ± 0.01a	0.01 ± 0.01a	0.01 ± 0.01a	0.01 ± 0.01a	0.01 ± 0.01a
Hexanoic acid	sour, cheesy	996	996	Acids	0.41 ± 0.28b	0.00c	0.00c	0.30 ± 0.19b	0.00c	0.33 ± 0.22b	0.00c	0.00c	0.00c	0.00c	0.88 ± 0.52a	0.00c
Ethyl hexanoate	fruity, pineapple	1000	999	Esters	9.47 ± 3.12b	7.09 ± 2.81de	8.18 ± 2.81c	7.94 ± 2.63c	9.66 ± 2.92ab	10.4 ± 3.81a	7.78 ± 2.12cd	4.75 ± 1.82f	4.41 ± 1.76f	3.24 ± 1.41g	5.13 ± 2.13f	6.47 ± 1.96e
Hexyl acetate	sweet, green apple, banana	1022	1022	Esters	0.55 ± 0.28c	0.05 ± 0.03f	0.20 ± 0.09d	0.97 ± 0.41a	0.19 ± 0.06d	0.89 ± 0.44a	0.00g	0.10 ± 0.06e	0.23 ± 0.11d	0.14 ± 0.07de	0.70 ± 0.29bc	0.81 ± 0.37ab
3-methylbutyl butanoate	apple skin	1067	1070	Esters	0.07 ± 0.04cd	0.03 ± 0.02d	0.13 ± 0.06bc	0.07 ± 0.04cd	0.57 ± 0.31b	0.19 ± 0.08c	0.00e	0.17 ± 0.08b	0.12 ± 0.04c	0.00e	0.70 ± 0.49a	0.06 ± 0.03d
Ethyl heptanoate	fruity, pineapple	1098	1082	Esters	0.11 ± 0.06cd	0.09 ± 0.04d	0.13 ± 0.06c	0.12 ± 0.05c	0.06 ± 0.03e	0.18 ± 0.11b	0.00f	0.07 ± 0.05de	0.10 ± 0.04d	0.37 ± 0.18a	0.14 ± 0.06bc	0.07 ± 0.04de
Phenylethyl alcohol	flowery, bread-like	1116	1114	Alcohols	13.92 ± 3.61b	7.75 ± 1.04f	5.56 ± 0.97g	12.34 ± 2.92c	8.41 ± 2.42ef	7.36 ± 1.52f	18.57 ± 3.84a	10.40 ± 2.88d	9.23 ± 1.82e	19.79 ± 4.19a	13.14 ± 4.01bc	10.18 ± 2.12d
Octanoic acid	oily, waxy	1190	1190	Acids	6.18 ± 3.82a	3.76 ± 1.84c	1.95 ± 0.74f	2.96 ± 1.34de	2.96 ± 1.27de	2.43 ± 0.92e	5.09 ± 2.24b	3.82 ± 1.95c	2.09 ± 0.81ef	3.09 ± 1.18cd	2.11 ± 0.76ef	2.45 ± 0.88e
Ethyl octanoate	waxy, musty	1197	1194	Esters	20.00 ± 2.88de	20.68 ± 2.97d	23.71 ± 4.84c	26.03 ± 5.72bc	28.09 ± 5.55b	33.02 ± 6.42a	21.2 ± 4.14d	16.3 ± 2.06f	18.66 ± 2.47e	14.12 ± 1.62g	20.91 ± 3.78d	25.97 ± 4.92bc
Hexyl pentanoate	fruity, appley, tropical, pear	1237	1235	Esters	0.05 ± 0.04e	0.14 ± 0.08d	0.78 ± 0.32b	0.00f	0.15 ± 0.08d	0.92 ± 0.49a	0.00f	0.06 ± 0.04e	0.48 ± 0.31c	0.00f	0.08 ± 0.06e	0.40 ± 0.26c
3-methylobutylhexanoate	fruity, waxy, leafy	1252	1253	Esters	0.07 ± 0.04e	0.37 ± 0.19c	0.51 ± 0.28ab	0.07 ± 0.05e	0.26 ± 0.12d	0.44 ± 0.26bc	0.00f	0.32 ± 0.18c	0.57 ± 0.31a	0.20 ± 0.11d	0.24 ± 0.16d	0.27 ± 0.18d
Phenylethyl acetate	flowery, honey-like	1257	1255	Esters	0.42 ± 0.15f	0.70 ± 0.31ef	0.41 ± 0.17f	0.75 ± 0.44ef	0.48 ± 0.28f	0.33 ± 0.18f	0.95 ± 0.62e	7.78 ± 4.13a	2.91 ± 1.07d	4.28 ± 1.41c	5.11 ± 1.63b	7.87 ± 3.85a
Decanoic acid	rotting citrus	1377	1373	Acids	2.35 ± 1.14bc	0.00f	1.04 ± 0.38d	0.00f	1.61 ± 0.78c	0.42 ± 0.28e	2.93 ± 1.15a	2.44 ± 1.06b	1.71 ± 0.91c	1.82 ± 0.93c	1.16 ± 0.56d	1.16 ± 0.49d
Ethyl (*E*)-dec-4-enoate	oleic, fruity, pineapple	1387	1379	Esters	0.00d	0.00d	0.00d	0.36 ± 0.17b	0.52 ± 0.24a	0.24 ± 0.14c	0.00d	0.00d	0.00d	0.32 ± 0.18bc	0.24 ± 0.16c	0.44 ± 0.21ab
Ethyl decanoate	appley, waxy, fruity	1395	1393	Esters	14.89 ± 2.85f	32.10 ± 6.08a	28.55 ± 5.91b	20.03 ± 4.83de	24.47 ± 4.06c	21.48 ± 5.34d	18.62 ± 3.84e	25.62 ± 4.84c	33.92 ± 6.41a	25.23 ± 4.11c	18.93 ± 3.91e	18.06 ± 4.02e
Pentyl octanoate	irisy, elderberry,	1447	1450	Esters	0.64 ± 0.28d	2.13 ± 0.92b	2.87 ± 1.15a	0.41 ± 0.15e	0.77 ± 0.31d	1.05 ± 0.64c	0.16 ± 0.09f	0.90 ± 0.49cd	2.19 ± 0.77b	0.48 ± 0.18e	0.36 ± 0.11e	0.36 ± 0.12e
3-methylbutyl octanoate	fruity, waxy	1449	1452	Esters	0.16 ± 0.09ab	0.13 ± 0.07b	0.20 ± 0.08a	0.00d	0.00d	0.10 ± 0.04bc	0.11 ± 0.04b	0.11 ± 0.05b	0.00d	0.17 ± 0.06a	0.11 ± 0.05b	0.07 ± 0.04c
Ethyl (2*E*,4*Z*)-deca,-4-dienoate	fruity, appley, pear	1479	1474	Esters	0.03 ± 0.02d	0.00e	0.00e	0.19 ± 0.09c	0.31 ± 0.19b	0.13 ± 0.07c	0.00e	0.00e	0.00e	0.50 ± 0.28a	0.24 ± 0.16bc	0.24 ± 0.15bc
(*Z*,*E*)-α-Farnesene	woody	1491	1495	Sesquiterpenes	0.05 ± 0.04d	0.09 ± 0.04bc	0.12 ± 0.05ab	0.02 ± 0.01e	0.08 ± 0.04c	0.06 ± 0.03cd	0.02 ± 0.01e	0.05 ± 0.03d	0.13 ± 0.06a	0.05 ± 0.04d	0.14 ± 0.08a	0.06 ± 0.04cd
(*E*,*E*)-α-Farnesene	woody, citrusy	1507	1505	Sesquiterpenes	0.37 ± 0.18cd	0.17 ± 0.09f	0.35 ± 0.18d	0.43 ± 0.22c	0.30 ± 0.13de	0.18 ± 0.10f	0.51 ± 0.31bc	0.78 ± 0.34a	0.32 ± 0.19d	0.26 ± 0.11e	0.19 ± 0.08f	0.19 ± 0.11f
Dodecanoic acid	oily	1575	1570	Acids	0.24 ± 0.16bc	0.51 ± 0.24a	0.17 ± 0.10cd	0.10 ± 0.04de	0.13 ± 0.06d	0.10 ± 0.06d	0.27 ± 0.13b	0.10 ± 0.05de	0.18 ± 0.09c	0.00f	0.00f	0.06 ± 0.04e
Ethyl dodecanoate	waxy, appley, fruity	1595	1595	Esters	13.68 ± 3.68a	14.02 ± 4.15a	13.47 ± 3.41a	6.61 ± 1.98f	6.61 ± 2.12f	6.74 ± 2.55ef	9.24 ± 2.81c	7.40 ± 2.63e	11.74 ± 2.88b	8.44 ± 2.35d	5.26 ± 1.54g	4.52 ± 1.01h

^1^ [17,18,19,20,21,22,23]; ^2^ I15—*Idared* apple variety cider fermented at 15 °C; IK15—*Idared* apple (80%) and *Konferencja* pear (20%) cider fermented at 15 °C; C15—*Cortland* apple variety cider fermented at 15 °C; CK15—*Cortland* apple (80%) and *Konferencja* pear (20%) cider fermented at 15 °C; G15—*Gala* apple variety cider fermented at 15 °C; GK15—*Gala* apple (80%) and *Konferencja* pear (20%) cider fermented at 15 °C; I20—*Idared* apple variety cider fermented at 20 °C; IK20—*Idared* apple (80%) and *Konferencja* pear (20%) cider fermented at 20 °C; C20—*Cortland* apple variety cider fermented at 15 °C; CK20—*Cortland* apple (80%) and *Konferencja* pear (20%) cider fermented at 20 °C; G20—*Gala* apple variety cider fermented at 20 °C; GK20—*Gala* apple (80%) and *Konferencja* pear (20%) varieties cider fermented at 20 °C. Values are expressed as means (n = 2) ± standard deviations. Mean values with different letters (a, b, c, d, e, f, g, h) within the same column are statistically different (*p*-value < 0.05).

**Table 3:** Concentrations of carbohydrates in juices.

Type of Beverage ^1^	Carbohydrates (*w/w*)
I	10.90 ± 0.15 ^c^
IK	11.70 ± 0.15 ^b^
C	11.70 ± 0.15 ^b^
CK	11.90 ± 0.20 ^b^
G	13.70 ± 0.12 ^a^
GK	13.90 ± 0.25 ^a^

^1^ I—*Idared* apple variety juice; IK—*Idared* apple (80%) and *Konferencja* pear (20%) juices mix; C—*Cortland* apple variety juice; CK—*Cortland* apple (80%) and *Konferencja* pear (20%) juices mix; G—*Gala* apple variety juice; GK—*Idared* apple (80%) and *Konferencja* pear (20%) juices mix. Values are expressed as mean (n = 3) ± standard deviation. Mean values with different letters (a, b, c,) within the same column are statistically different (*p*-value < 0.05).

**Table 4:** Concentrations of carbohydrates and ethanol and degree of attenuation in ciders.

Type of Beverage ^1^	Ethanol (*v/v*)	Carbohydrates (*w/w*)	Degree of Attenuation (%)
I15	5.19 ± 0.06^e^	3.95 ± 0.05^b^	63.76^f^
IK15	4.88 ± 0.13^f^	3.15 ± 0.07^def^	73.08^d^
C15	4.12 ± 0.09^h^	4.38 ± 0.06^a^	60.54^g^
CK15	5.18 ± 0.11^e^	2.97 ± 0.04^ef^	75.04^c^
G15	6.38 ± 0.08^c^	3.90 ± 0.09^b^	71.53^e^
GK15	6.36 ± 0.08^c^	3.82 ± 0.08^bc^	72.52^d^
I20	4.92 ± 0.12^f^	2.23 ± 0.05^g^	86.70^a^
IK20	5.66 ± 0.20^d^	2.92 ± 0.10^ef^	75.04^c^
C20	4.57 ± 0.05^g^	3.08 ± 0.07^ef^	72.25^de^
CK20	5.61 ± 0.09^d^	2.80 ± 0.06^f^	76.47^b^
G20	6.56 ± 0.14^b^	3.24 ± 0.07^de^	76.35^b^
GK20	6.79 ± 0.04^a^	3.49 ± 0.04^cd^	74.89^c^

^1^ I15—*Idared* apple variety cider fermented at 15 °C; IK15—*Idared* apple (80%) and *Konferencja* pear (20%) cider fermented at 15 °C; C15—*Cortland* apple variety cider fermented at 15 °C; CK15—*Cortland* apple (80%) and *Konferencja* pear (20%) cider fermented at 15 °C; G15—*Gala* apple variety cider fermented at 15 °C; GK15—*Gala* apple (80%) and *Konferencja* pear (20%) cider fermented at 15 °C; I20—*Idared* apple variety cider fermented at 20 °C; IK20—*Idared* apple (80%) and *Konferencja* pear (20%) varieties cider fermented at 20 °C; C20—*Cortland* apple variety cider fermented at 15 °C; CK20 *Cortland* apple (80%) and *Konferencja* pear (20%) cider fermented at 20 °C; G20—*Gala* apple variety cider fermented at 20 °C; GK20—*Gala* apple (80%) and *Konferencja* pear (20%) varieties cider fermented at 20 °C. Values are expressed as mean (n = 3) ± standard deviation. Mean values with different letters (a, b, c, d, e, f, g) within the same column are statistically different (*p*-value < 0.05).
